# Supplementary material for: Integrated interfacial design of covalent organic framework photocatalysts to promote hydrogen evolution from water
Source: Nat Commun. 2023 Jan 19;14:329. doi: 10.1038/s41467-023-35999-y (PMC9852592; doi:10.1038/s41467-023-35999-y)
Supplement: Supplementary file 1 — Supplementary Information [file 41467_2023_35999_MOESM1_ESM.pdf]

Supplementary Information

Integrated interfacial design of covalent organic framework photocatalysts to promote hydrogen evolution from water

Ting He[1], Wenlong Zhen[2], Yongzhi Chen[1], Yuanyuan Guo[3], Zhuoer Li[1,4], Ning Huang[5], Zhongping Li[1], Ruoyang Liu[1], Yuan Liu[1], Xu Lian[1], Can Xue[2], Tze Chien Sum[3], Wei Chen[1] & Donglin Jiang[1,4]*

[1]Department of Chemistry, Faulty of Science, National University of Singapore, 3 Science Drive 3, Singapore 117543, Singapore

[2]School of Materials Science and Engineering, Nanyang Technological University, 50 Nanyang Avenue, 639798 Singapore

[3]Division of Physics and Applied Physics, School of Physical and Mathematical Sciences, Nanyang Technological University, 21 Nanyang Link, Singapore 637371, Singapore

[4]Joint School of National University of Singapore and Tianjin University, International Campus of Tianjin University, Fuzhou, 350207, China

[5]MOE Key Laboratory of Macromolecular Synthesis and Functionalisation, Department of Polymer Science and Engineering, Zhejiang University, Hangzhou 310027, China

*Corresponding Author: Prof. Donglin Jiang (chmjd@nus.edu.sg)

Table of Contents

1. Materials and Methods--- 2

2. Supplementary Figures---6

3. Supplementary Tables---28

4. Supplementary References--41

1. Materials and Methods

Characterization

Powder X-ray diffraction (PXRD) measurement was conducted on a Bruker D8 Focus Powder X-ray Diffractometer using Cu Kα radiation (40 kV, 40 mA) at room temperature by depositing powder on polytetrafluoroethylene substrate, from $2\theta = 2°$ to 30° with 0.01° increment. Fourier Transform Infrared (FT IR) spectra were performed on a Bruker VERTEX 80v spectrometer. ^1H NMR spectra were recorded on a Bruker AVANCE I 500 MHz NMR spectrometer. Solid-state NMR experiments were conducted on a Bruker Avance III HD 600 MHz wide-bore NMR spectrometer. Nitrogen sorption isotherms were recorded at 77 K with a Micromeritics Instrument Corporation model 3Flex surface characterization analyzer. The surface area was calculated by the Brunauer–Emmett–Teller (BET) method. The pore size and pore volume were calculated by non-local density functional theory (NLDFT) model from the sorption curve. Thermogravimetric (TG) analysis was conducted on a Discovery TGA at a constant heating rate of 10 °C min^{-1} from room temperature to 800 °C under nitrogen. Scanning electron microscopy (SEM) measurement were performed with a JEOL JSM-6701F microscope. The metal content was determined by inductively coupled plasma mass spectrometry (ICP-MS) on an Agilent 7700x analyzer. The contact angle was recorded by LAUDA Scientific Surface Analyzer (LSA/OAS-100/200/60). Water sorption analysis was conducted by Quantachrome Instruments Autosorb-iQ.

Chemicals

o-Dichlorobenzene (*o*-DCB), *n*-butanol (*n*-BuOH), 1,4-dioxane, ethanol (EtOH), methanol (MeOH), tetrahydrofuran (THF), hexane, ethyl acetate, dichloromethane and acetone were purchased from TCI. Lactic acid, *m*-CPBA, 2,5-dimethylpyrazine, acetic anhydride, 1-bromo-2-(2-methoxyethoxy)ethane, CH_3ONa, activated MnO_2, terephthalaldehyde, $(NH_4)_6Mo_7O_{24}•4H_2O$ and ammonium polysulfide solution were purchased from Sigma-Aldrich. Zinc-5,10,15,20-tetrakis(*p*-tetraphenylamino) porphyrin (ZnP)[1,2], pyrazine-2,5-dialdehyde[3], 2,5-dihydroxyterephthalaldehyde[4,5] and $(NH_4)_2Mo_3S_{13}•nH_2O$ were synthesized by the literature procedures[6].

Temperature-dependent photoluminescence spectra

Temperature-dependent photoluminescence spectra were recorded by Edinburgh Instruments

(FLS980) to determine the exciton binding energy (E_b) of COFs. The intensity of PL decreases when the temperature increases. The corresponding E_b is calculated through fitting the intensity data with Arrhenius equation, $I(T)=I_0/(1+A\exp(-E_b/k_BT))$[7].

Transient absorption spectroscopy

Transient absorption spectroscopy was conducted with a HELIOS femtosecond transient absorption spectrometer (Ultrafast Systems, LLC). The 400 nm (3.1 eV) pump pulses were generated by frequency doubling of the 800 nm fundamental output from the regenerative amplifier (Libra, 1 kHz, 50 fs) with a BBO crystal. The probe pulse used was a visible white light continuum (400 – 850 nm) generated by focusing the 800 nm fundamental output from the regenerative amplifier into a 2 mm sapphire crystal. The probe white light beam was passed through a 750 nm short-pass filter to eliminate any residual 800 nm fundamental components to prevent any strong secondary photoexcitation of the sample. The solution samples were dissolved in a quartz cuvette (2 mm path length) and constantly stirred using a magnetic stirrer during the experiments to prevent thermal lensing and samples degradation.

Apparent Quantum Efficiency (AQE)

AQE was measured under the same conditions by using the same Xenon lamp equipped with different bandpass filters of 420, 500, 600 and 700 nm. The intensity of irradiation light was measured by solar power meter (Newport, Model 1918-R). The average amount of H_2 was measured to calculate AQE in according to the following equation.

$$\eta_{AQY} = \frac{N_e}{N_p}\times 100\% = \frac{2\times M\times N_A}{\frac{E_{total}}{E_{photon}}}\times 100\% = \frac{2M\times N_A}{S\times P\times t}\times 100\% = \frac{2\times M\times N_A\times h\times c}{S\times P\times t\times\lambda}\times 100\%$$

M is the amount of H_2 (mol), N_A is Avogadro constant (6.022×10^{23} mol^{-1}), h is the Planck constant (6.626×10^{-34} J S), c is the speed of light (3×10^{8} m s^{-1}), S is the irradiation area (cm^2), P is the intensity of irradiation light (W cm^{-2}), t is the photoreaction time (s), λ is the wavelength of the monochromatic light (nm).

Photoelectrochemical measurement

Photoelectrochemical measurement were conducted using a three-electrode CHI 650D electrochemical workstation. The counter electrode was a Pt sheet and the reference electrode was Ag/AgCl. For the preparation of the working electrodes, the as-synthesized samples (2 mg) were added into 10 μL Nafion and 1 mL ethanol mixed solution, then the catalyst suspension (200 μL) were dropped onto fluorinated tin oxide glass (2 cm × 2 cm), forming a film after drying naturally for 24 h. An aqueous Na_2SO_4 solution (0.5 M) was used as the electrolyte. The photoelectrochemical activity was measured using the linear-sweep voltammetry method with a scanning rate of 10 mV $s^{-1}$and a bias potential of 0.01 mV. A 300-W Xe lamp equipped with a 420 nm cutoff filter was employed for the irradiation, and a shutter was used to modulate the light and dark conditions during the test.

Electrochemical measurement

Cyclic voltammetry was performed using ALS/H CH Instruments Electrochemical Analyzer Model 610B with a three-electrode electrochemical cell. The scan rate was 0.1 V s^{-1}. The electrolyte was anhydrous acetonitrile with tetrabutylammonium hexafluorophosphate (0.1 M). The counter electrode was platinum wire. The reference electrode was based on $Ag/AgNO_3$ (10^{-2} M in CH_3CN). The working electrode was a carbon glass electrode. The experiments were calibrated with the standard ferrocene/ferrocenium (Fc) redox system and assumption that the energy level of Fc is 4.8 eV below vacuum.

Turnover frequency (TOF)

TOF is a kinetic-dependent parameter, which was calculated according to the following equation:

$$TOF = \frac{M_{H_2}}{M_{Mo}}$$

M_{H2} is the amount of H_2 molecules evolved in unit time (mmol g^{-1} h^{-1}); M_{Mo} is the amount of Mo (mmol g^{-1}).

Hydrolysis of ZnP-Pz-DHTP-COF

A ZnP-Pz-DHTP-COF sample (100 mg) dispersed in a mixture of aqueous KOH solution (4 M)

and THF (1/2 in vol, 30 mL) was refluxed for 3 days. The mixture was filtrated and added with HCl (4 M, 10 mL). The greenish zinc-porphyrin was removed by filtration. The solid was collected by drying under vacuum and submitted to ^{1}H NMR spectroscopy in $CDCl_3$.

2. Supplementary Figures

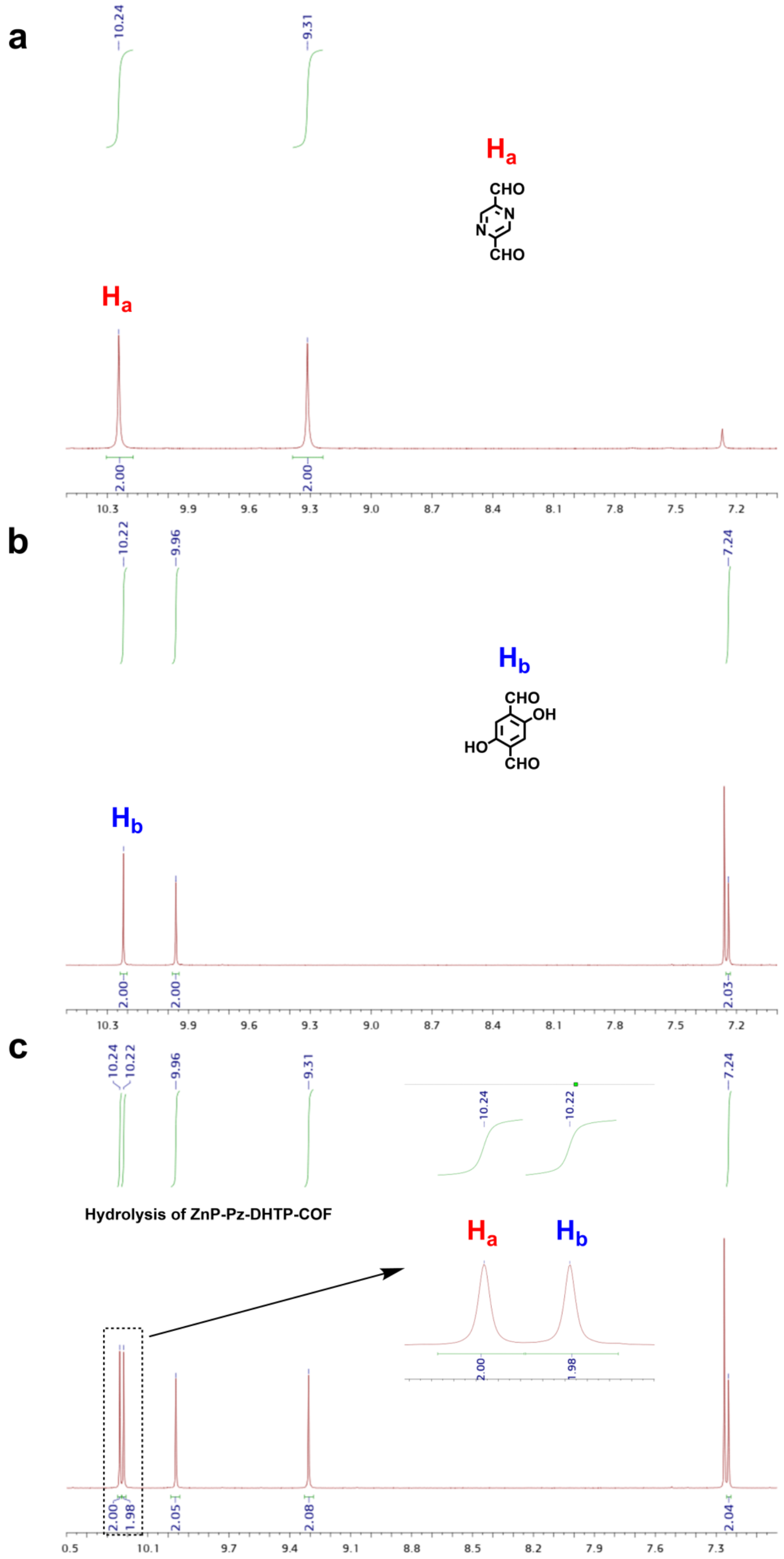

Supplementary Figure 1 | ¹H NMR spectra. a, ¹H NMR spectrum of monomer pyrazine-2,5-dialdehyde. **b,** ¹H NMR spectrum of monomer 2,5-dihydroxyterephthalaldehyde. **c,** ¹H NMR spectrum of hydrolysed ZnP-Pz-DHTP-COF.

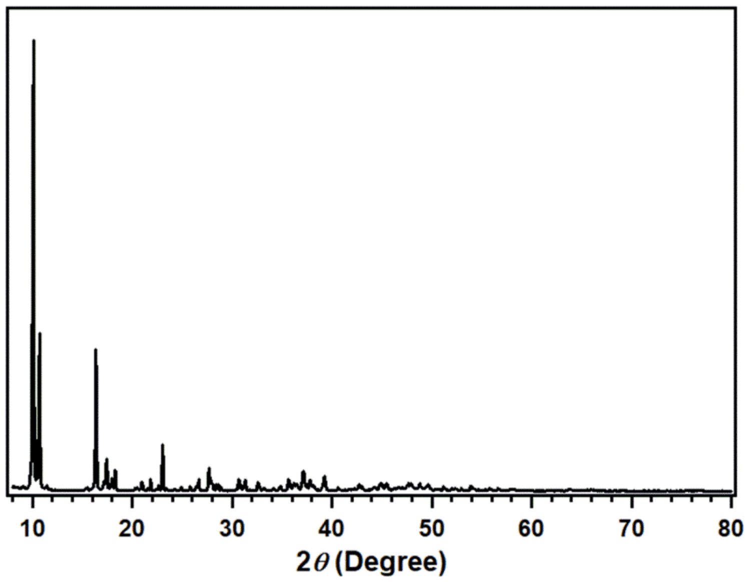

Supplementary Figure 2 | PXRD pattern. PXRD pattern of $(NH_4)_2Mo_3S_{13}$.

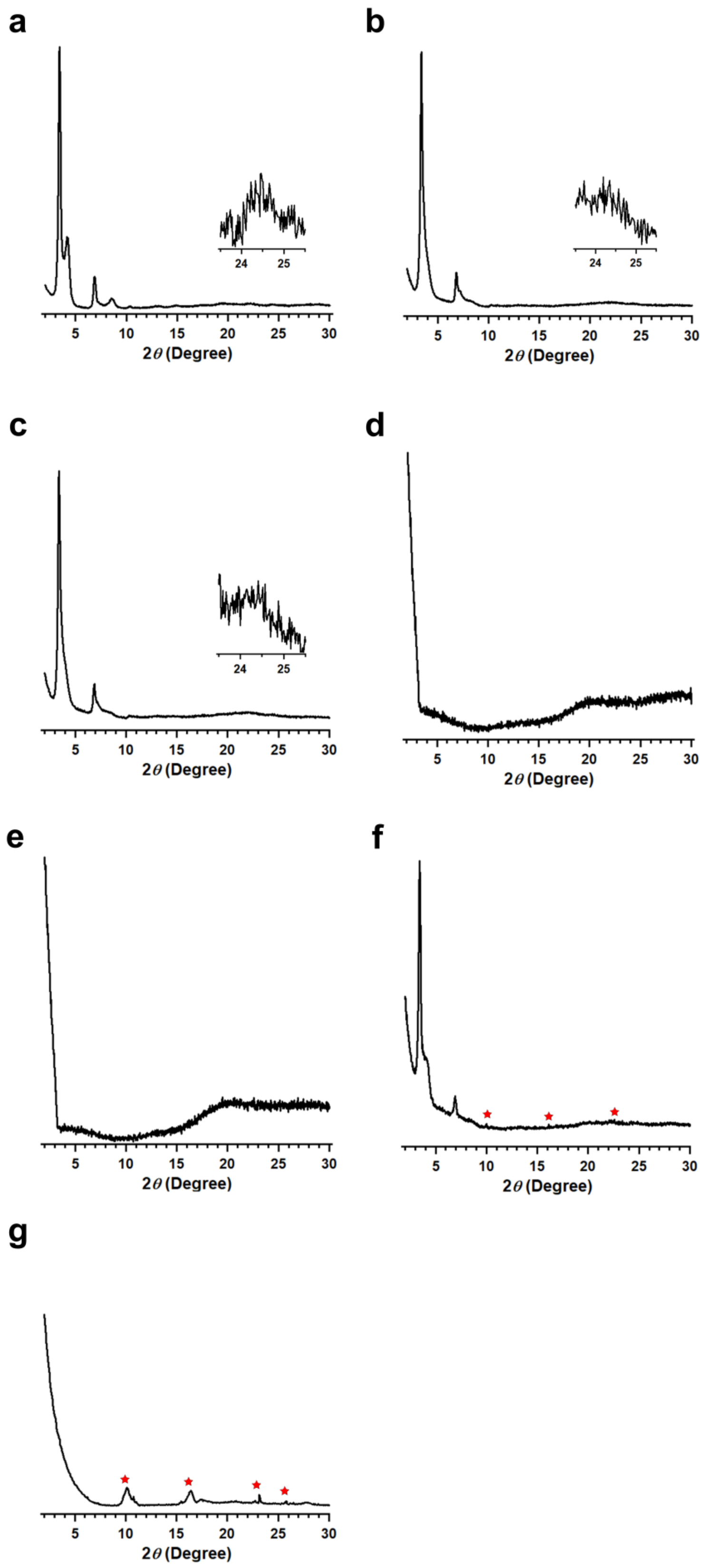

Supplementary Figure 3 | PXRD patterns. a-g, The PXRD patterns of (a) ZnP-Pz-COF, (b) ZnP-TP-DHTP-COF, (c) ZnP-TP-PEO-COF, (d) ZnP-Pz-DHTP-POP, (e) ZnP-Pz-PEO-POP, (f) $[Mo_3S_{13}]^{2-}$/ZnP-TP-PEO-COF and (g) $[Mo_3S_{13}]^{2-}$@ZnP-Pz-PEO-POP.

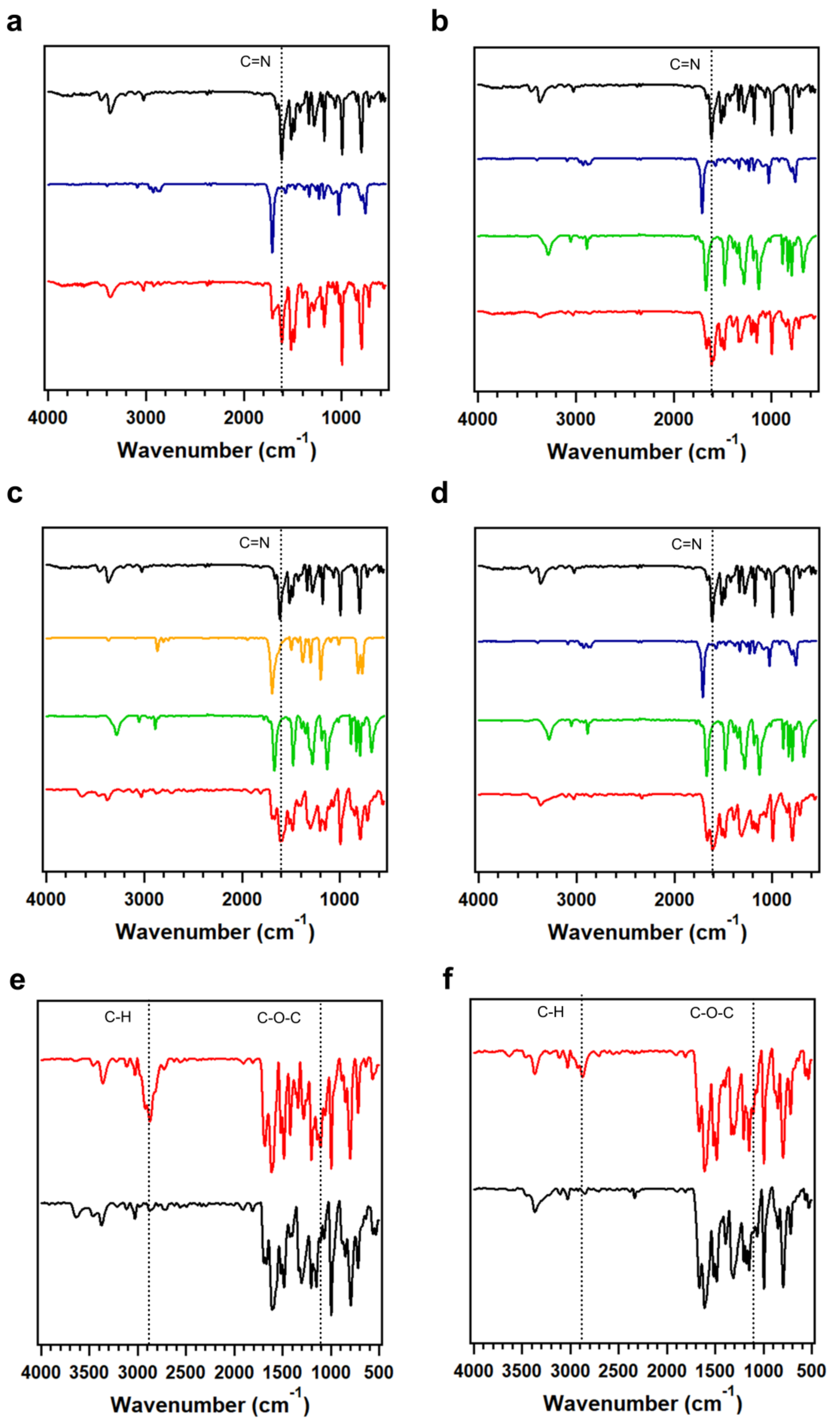

Supplementary Figure 4 | FT IR spectra. a-d, FT IR spectra of (a) ZnP-Pz-COF, (b) ZnP-Pz-DHTP-COF, (c) ZnP-TP-DHTP-COF and (d) ZnP-Pz-DHTP-POP (ZnP, black curve; PzDA, blue curve; BDA, orange curve; DHTA, green curve; COFs or polymer, red curve). **e** and **f,** FT IR spectra of (e) ZnP-TP-PEO-COF (red curve) and (f) ZnP-Pz-PEO-POP (red curve). Black curves in **e** and **f** are ZnP-TP-DHTP-COF and ZnP-Pz-DHTP-POP, respectively.

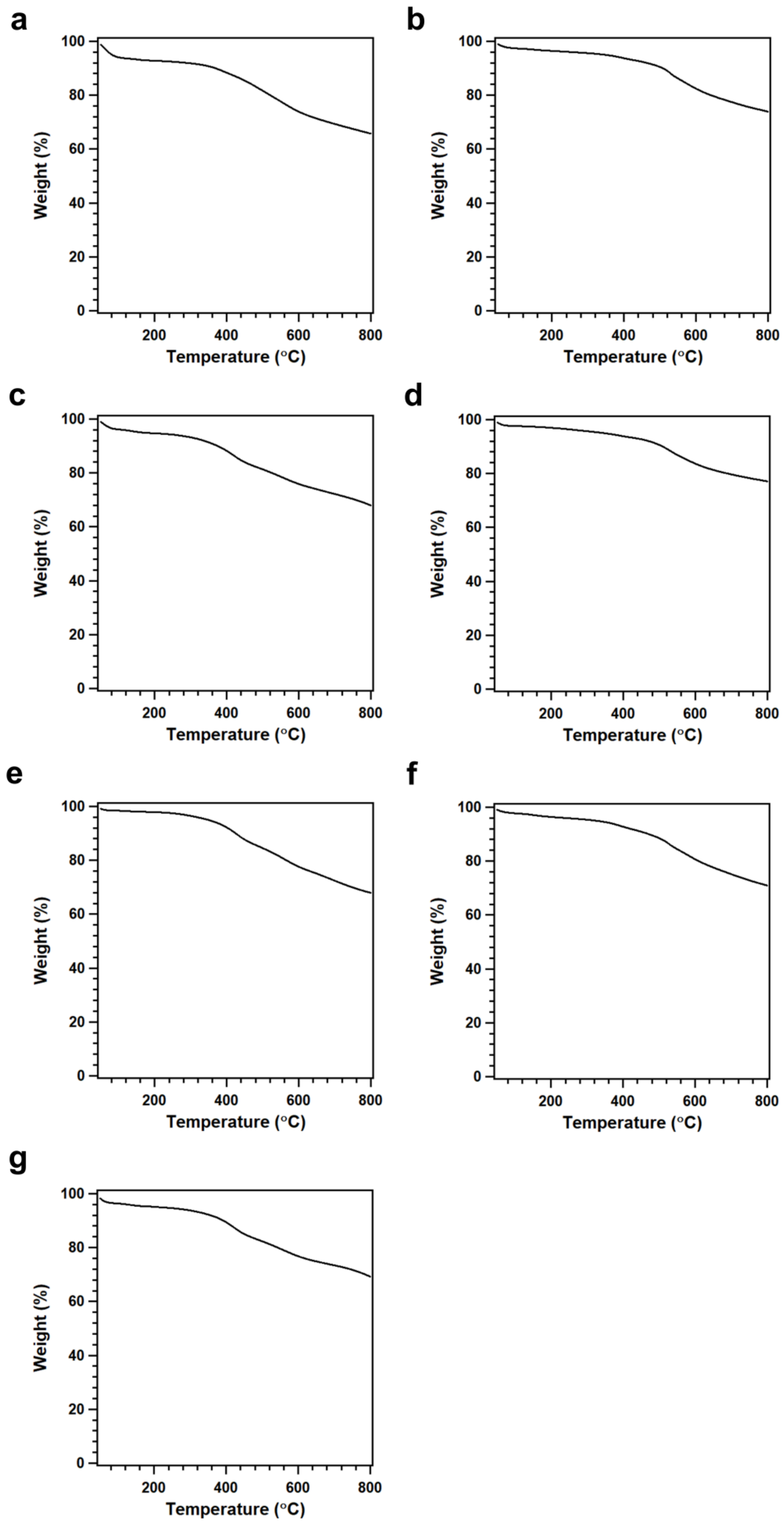

Supplementary Figure 5 | Thermogravimetric analysis curves. a–g, TGA curves of (a) ZnP-Pz-COF, (b) ZnP-Pz-DHTP-COF, (c) ZnP-Pz-PEO-COF, (d) ZnP-TP-DHTP-COF, (e) ZnP-TP-PEO-COF, (f) ZnP-Pz-DHTP-POP and (g) ZnP-Pz-PEO-POP.

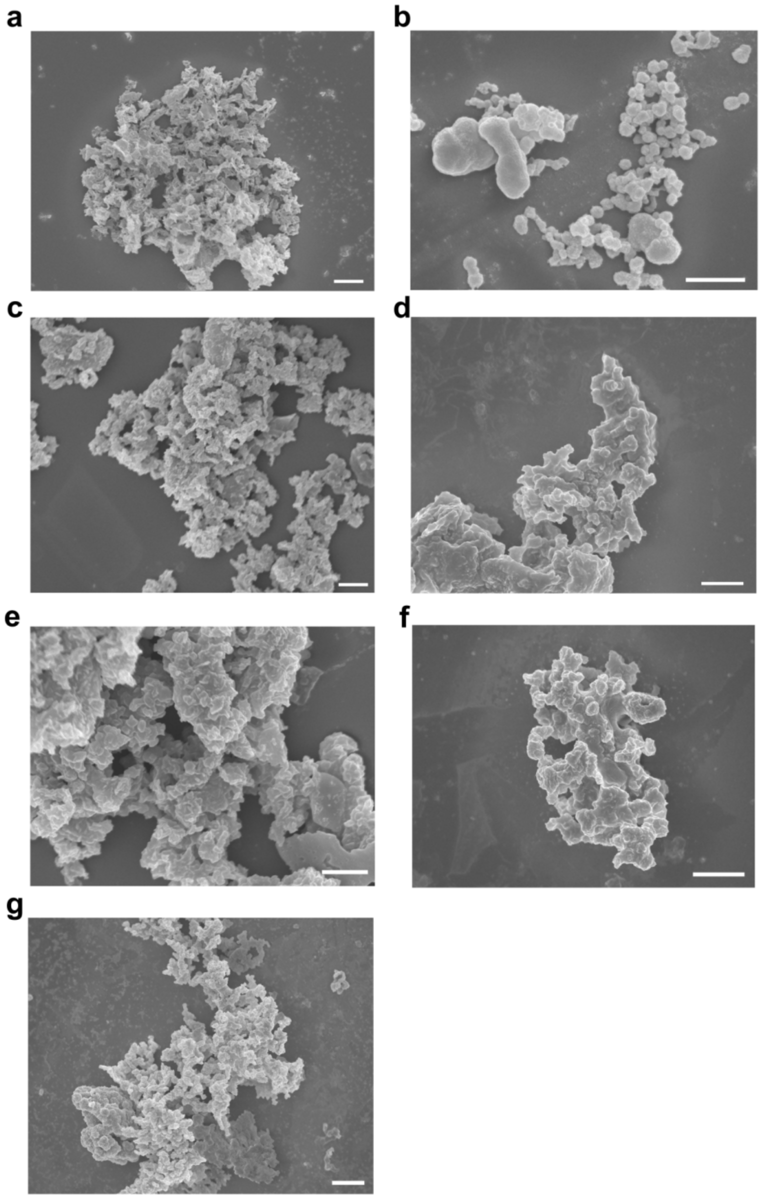

Supplementary Figure 6 | Scanning electron microscope images. a-g, SEM images of (a) ZnP-Pz-DHTP-COF, (b) ZnP-Pz-COF, (c) ZnP-TP-DHTP-COF, (d) ZnP-Pz-DHTP-POP, (e) ZnP-Pz-PEO-COF, (f) ZnP-TP-PEO-COF and (g) ZnP-Pz-PEO-POP (scale bar, 1 μm).

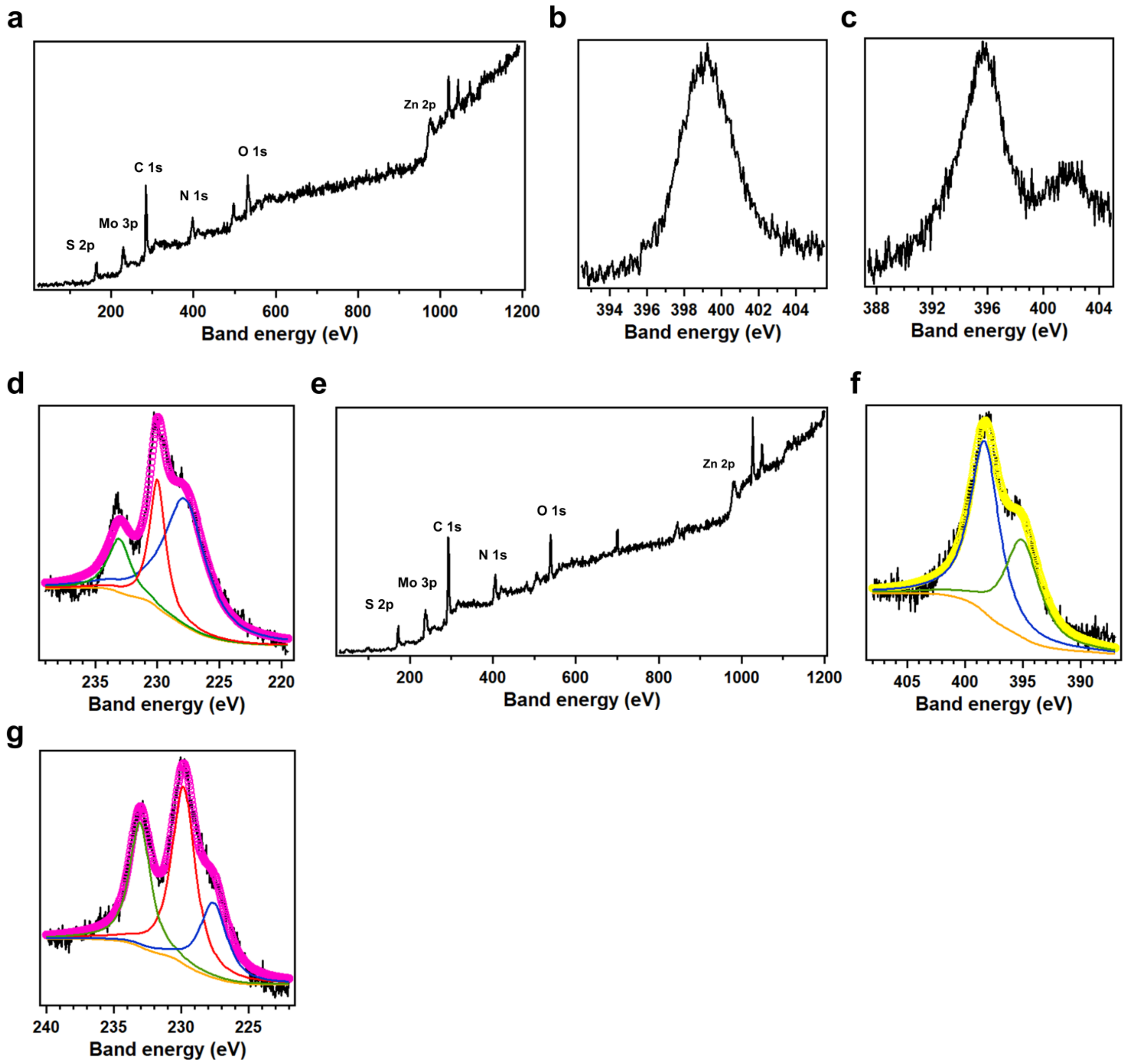

Supplementary Figure 7 | X-ray photoelectron spectroscopy spectra. a, The XPS survey spectrum of $[Mo_3S_{13}]^{2-}$@ZnP-Pz-PEO-COF. **b** and **c,** XPS N *1s* spectra of (b) ZnP-Pz-PEO-COF and (c) $(NH_4)_2Mo_3S_{13}$. **d,** XPS Mo *3d* spectrum of $[Mo_3S_{13}]^{2-}$@ZnP-Pz-PEO-COF (green curve: Mo $3d_{3/2}$; red curve: Mo $3d_{5/2}$; blue curve: S *2s*)[6]. **e,** The XPS survey spectrum of $[Mo_3S_{13}]^{2-}$/ZnP-TP-PEO-COF. **f,** XPS N *1s* spectrum of $[Mo_3S_{13}]^{2-}$/ZnP-TP-PEO-COF. **g,** XPS Mo *3d* spectrum of $[Mo_3S_{13}]^{2-}$/ZnP-TP-PEO-COF (green curve: Mo $3d_{3/2}$; red curve: Mo $3d_{5/2}$; blue curve: S *2s*)[6].

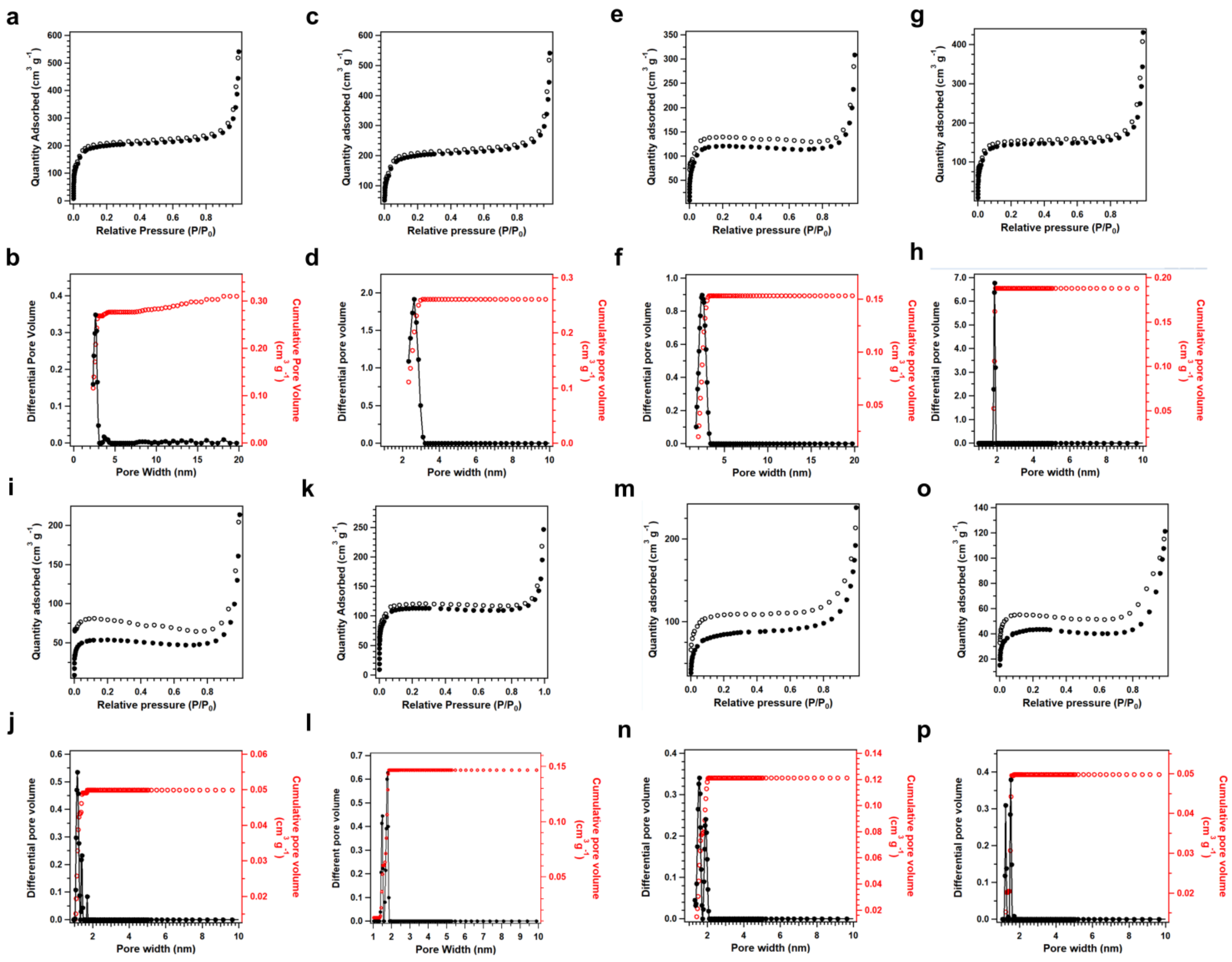

Supplementary Figure 8 | Nitrogen sorption curves, pore size, and pore size distribution profiles. a, c, e, g, i, k, m and **o,** Nitrogen sorption curves of (a) ZnP-Pz-COF, (c) ZnP-TP-DHTP-COF, (e) ZnP-Pz-DHTP-POP, (g) ZnP-TP-PEO-COF, (i) ZnP-Pz-PEO-POP, (k) $[Mo_3S_{13}]^{2-}$@ZnP-Pz-PEO-COF, (m) $[Mo_3S_{13}]^{2-}$/ZnP-TP-PEO-COF and (o) $[Mo_3S_{13}]^{2-}$@ZnP-Pz-PEO-POP. **b, d, f, h, j, l, n** and **p,** pore size distribution and pore volume curves of (b) ZnP-Pz-COF, (d) ZnP-TP-DHTP-COF, (f) ZnP-Pz-DHTP-POP, (h) ZnP-TP-PEO-COF, (j) ZnP-Pz-PEO-POP, (l) $[Mo_3S_{13}]^{2-}$@ZnP-Pz-PEO-COF, (n) $[Mo_3S_{13}]^{2-}$/ZnP-TP-PEO-COF and (p) $[Mo_3S_{13}]^{2-}$@ZnP-Pz-PEO-POP.

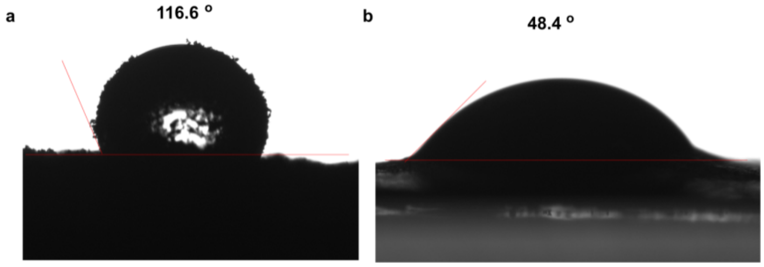

Supplementary Figure 9 | Water contact angle images. a, Image of contact angle measurement of $[Mo_3S_{13}]^{2-}$@ZnP-Pz-COF. **b,** Image of contact angle measurement of $[Mo_3S_{13}]^{2-}$@ZnP-Pz-DHTP-COF.

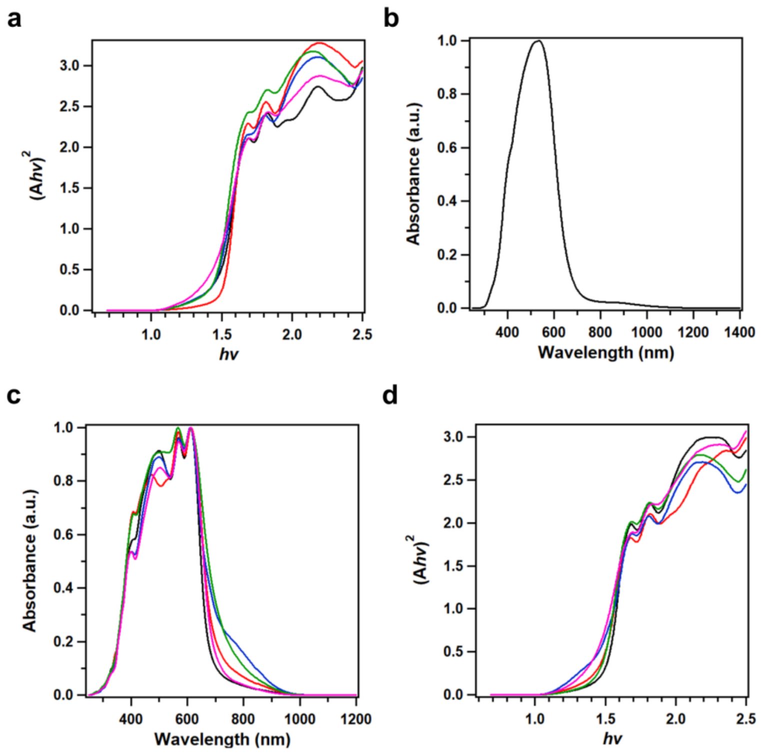

Supplementary Figure 10 | Solid-state UV-Vis diffuse reflection absorption spectra and Kubelka-Munk-transformed reflectance spectra. a, Band gaps of ZnP-Pz-COF (black curve), ZnP-Pz-DHTP-COF (red curve), ZnP-Pz-PEO-COF (blue curve), ZnP-TP-PEO-COF (green curve) and ZnP-Pz-PEO-POP (purple curve) determined by the Kubelka-Munk-transformed reflectance spectra. **b,** Solid-state UV–Vis diffuse reflection absorption spectrum of $(NH_4)_2Mo_3S_{13}$. **c,** Solid-state UV–Vis diffuse reflection absorption spectra. **d,** Band gaps of $[Mo_3S_{13}]^{2-}$@ZnP-Pz-COF (black curve), $[Mo_3S_{13}]^{2-}$@ZnP-Pz-DHTP-COF (red curve), $[Mo_3S_{13}]^{2-}$@ZnP-Pz-PEO-COF (blue curve), $[Mo_3S_{13}]^{2-}$/ZnP-TP-PEO-COF (green curve) and $[Mo_3S_{13}]^{2-}$@ZnP-Pz-PEO-POP (purple curve).

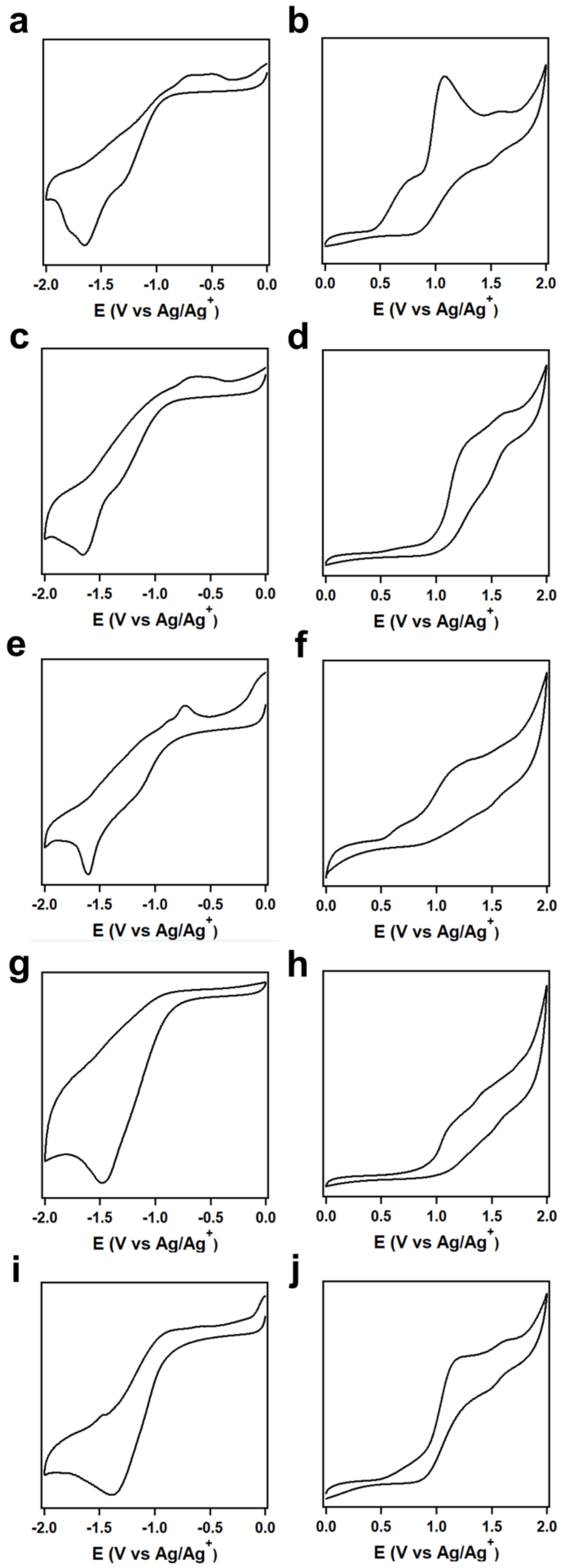

Supplementary Figure 11 | Cyclic voltammetry curves. a–j, Cyclic voltammetry results of (a, b) ZnP-Pz-COF (c, d) ZnP-Pz-DHTP-COF, (e, f) ZnP-Pz-PEO-COF, (g, h) ZnP-TP-PEO-COF and (i, j) ZnP-Pz-PEO-POP.

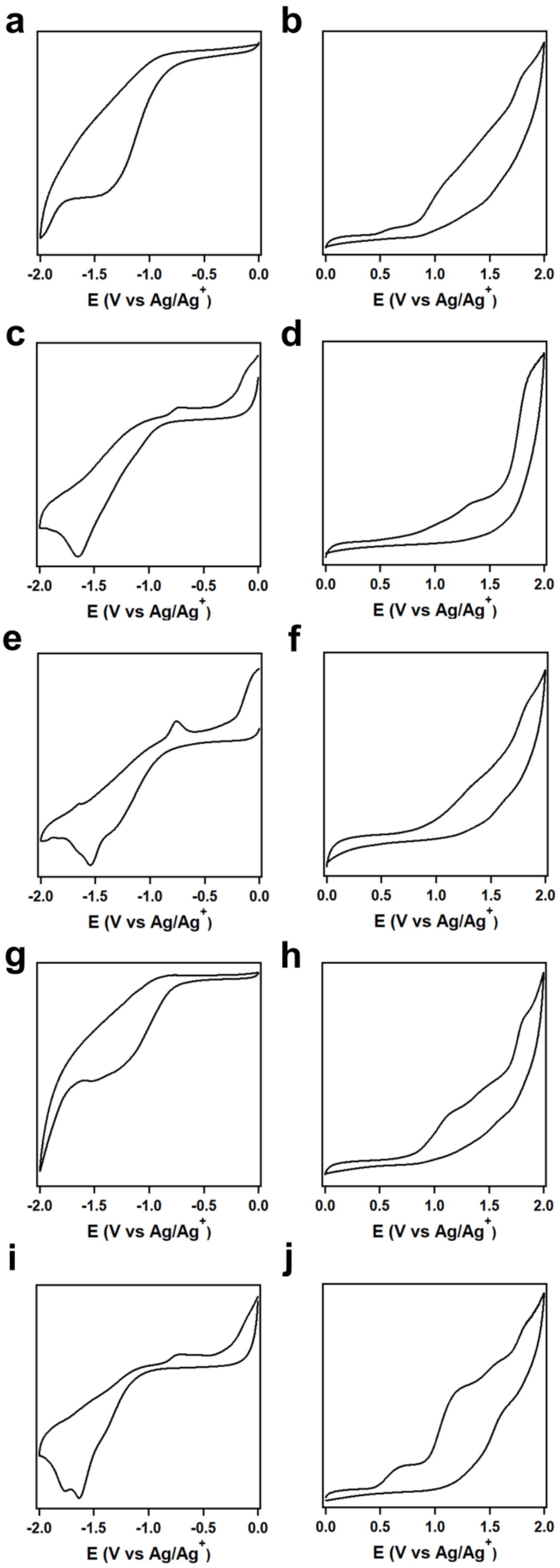

Supplementary Figure 12 | Cyclic voltammetry curves. a–j, Cyclic voltammetry results of (a, b) $[Mo_3S_{13}]^{2-}$@ZnP-Pz-COF, (c, d) $[Mo_3S_{13}]^{2-}$@ZnP-Pz-DHTP-COF, (e, f) $[Mo_3S_{13}]^{2-}$@ZnP-Pz-PEO-COF, (g, h) $[Mo_3S_{13}]^{2-}$/ZnP-TP-PEO-COF and (i, j) $[Mo_3S_{13}]^{2-}$@ZnP-Pz-PEO-POP.

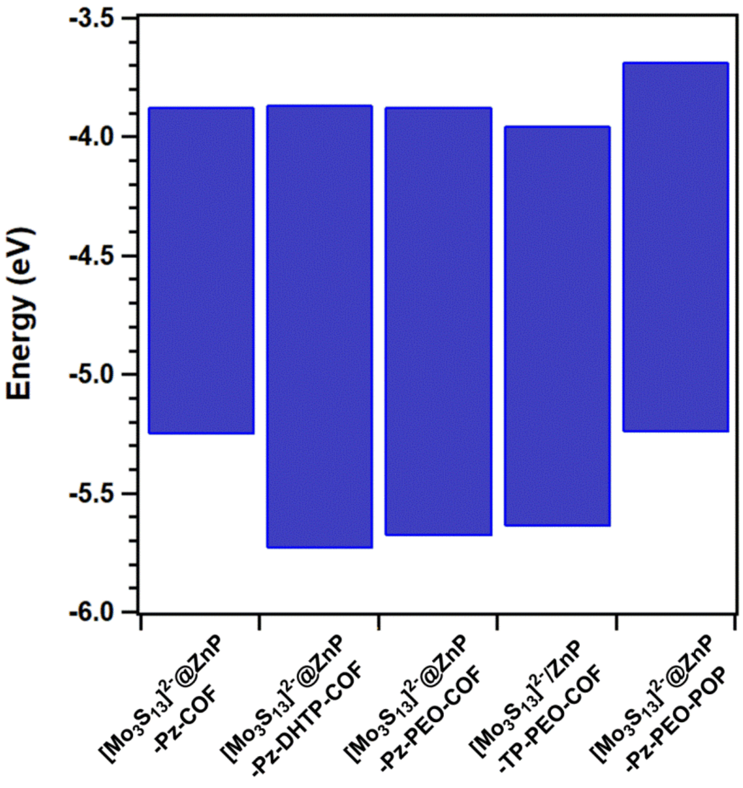

Supplementary Figure 13 | Band structures. Band structures of $[Mo_3S_{13}]^{2-}$@ZnP-Pz-COF, $[Mo_3S_{13}]^{2-}$@ZnP-Pz-DHTP-COF, $[Mo_3S_{13}]^{2-}$@ZnP-Pz-PEO-COF, $[Mo_3S_{13}]^{2-}$/ZnP-TP-PEO-COF and $[Mo_3S_{13}]^{2-}$@ZnP-Pz-PEO-POP.

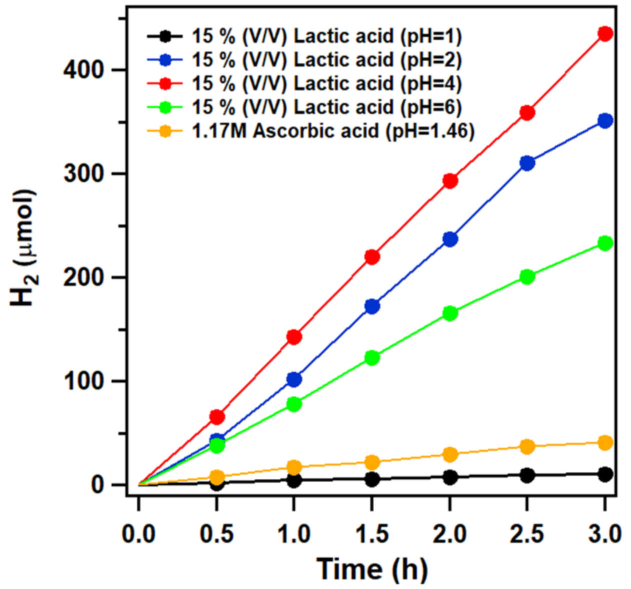

Supplementary Figure 14 | Photocatalytic activity. Hydrogen evolution of $[Mo_3S_{13}]^{2-}$@ZnP-Pz-PEO-COF with different sacrificial donor under different pH value over 3 h under visible light.

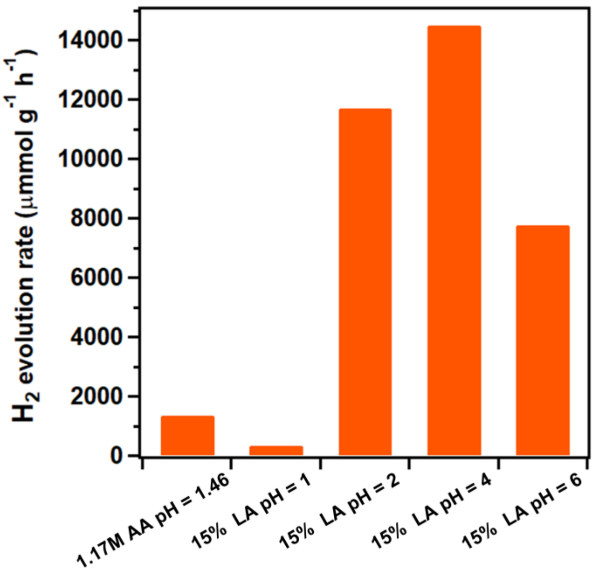

Supplementary Figure 15 | Photocatalytic activity. Hydrogen evolution rates of $[Mo_3S_{13}]^{2-}$ @ZnP-Pz-PEO-COF with different sacrificial donor under different pH values.

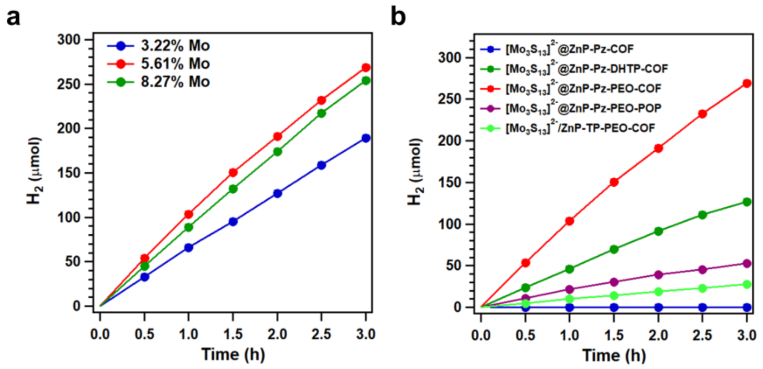

Supplementary Figure 16 | Photocatalytic activity. a, Hydrogen evolution of $[Mo_3S_{13}]^{2-}$@ZnP-Pz-PEO-COF with different contents of Mo cluster over 3 h in the presence of 15 vol% lactic acid under visible light. **b,** Hydrogen evolution of different samples monitored over 3 h in the presence of 15 vol% lactic acid under visible light.

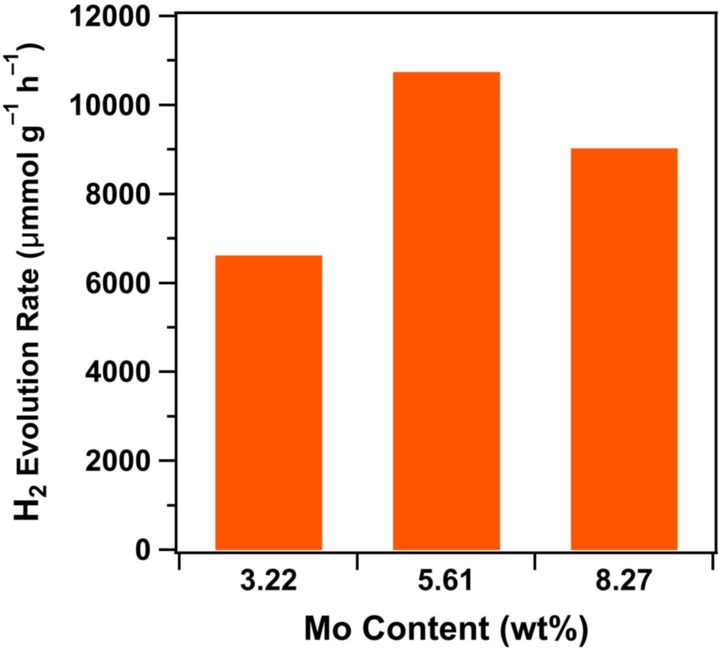

Supplementary Figure 17 | Photocatalytic activity. Hydrogen evolution rates of $[Mo_3S_{13}]^{2-}$@ZnP-Pz-PEO-COF with different $[Mo_3S_{13}]^{2-}$ contents.

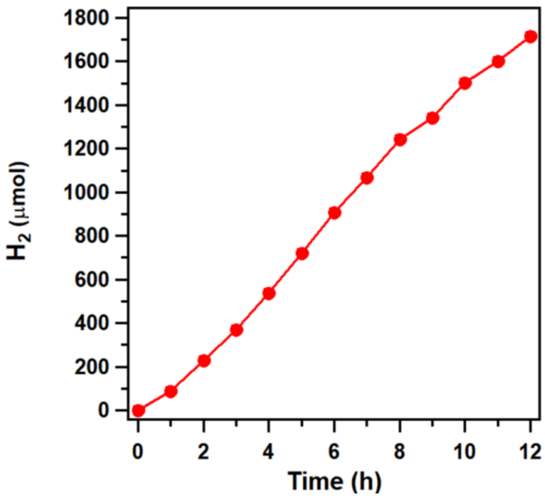

Supplementary Figure 18 | Stability. Long-term stability of $[Mo_3S_{13}]^{2-}$@ZnP-Pz-PEO-COF upon 12-h photocatalytic operation under continuous irradiation ($\lambda > 420$ nm).

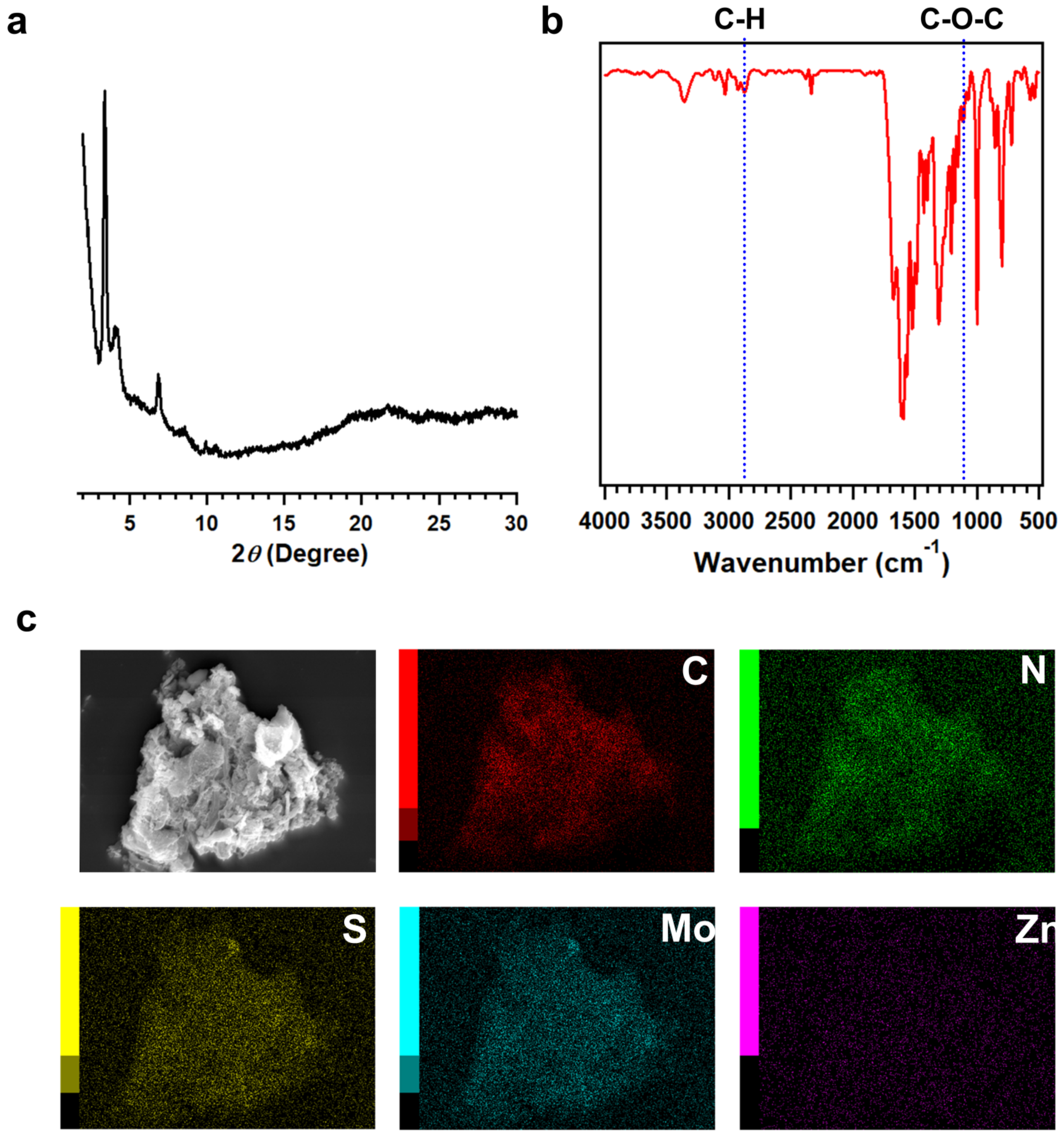

Supplementary Figure 19 | Characterizations after long-term photocatalytic reaction. a–c, (a) PXRD pattern, (b) FT IR spectrum and (c) EDX-element mapping of $[Mo_3S_{13}]^{2-}$@ZnP-Pz-PEO-COF after continuous photocatalysis.

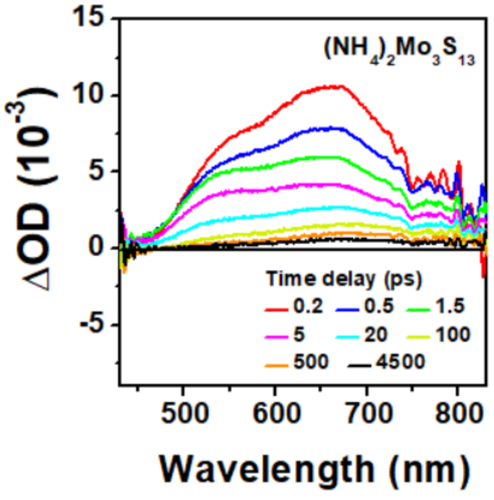

Supplementary Figure 20 | Femtosecond transient absorption spectra. Femtosecond transient absorption measurement of $(NH_4)_2Mo_3S_{13}$.

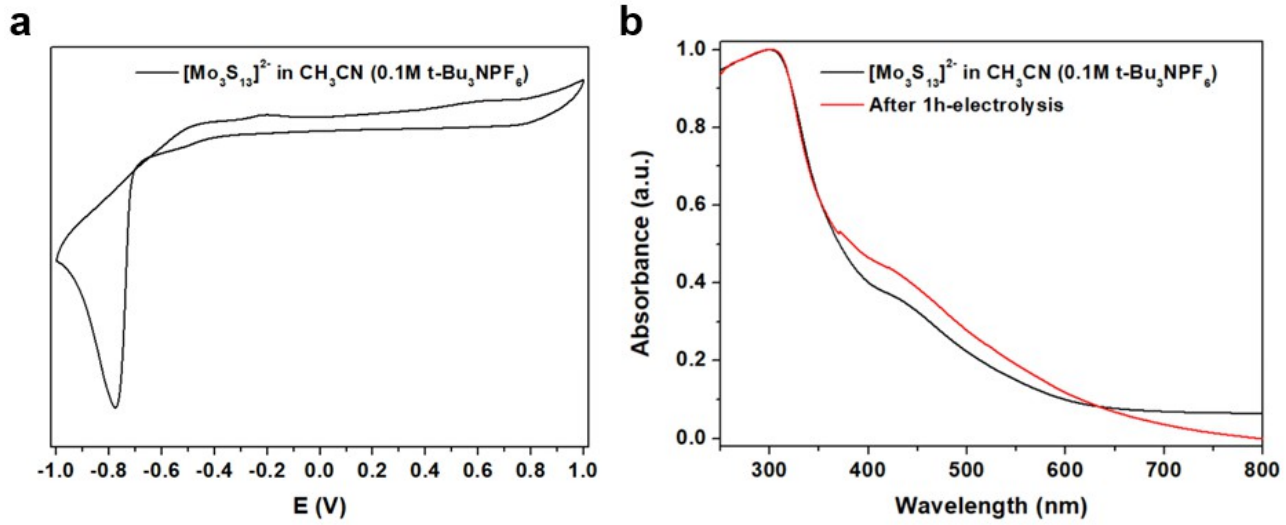

Supplementary Figure 20 | Photoelectrichemical experiments. a, Cyclic voltammetry curve of $(NH_4)_2Mo_3S_{13}$ in CH_3CN (0.1 M *t*-Bu_4NPF_6). **b**, UV-Vis spectra of $(NH_4)_2Mo_3S_{13}$ in CH_3CN before and after 1h electrolysis at –0.75V under Ar.

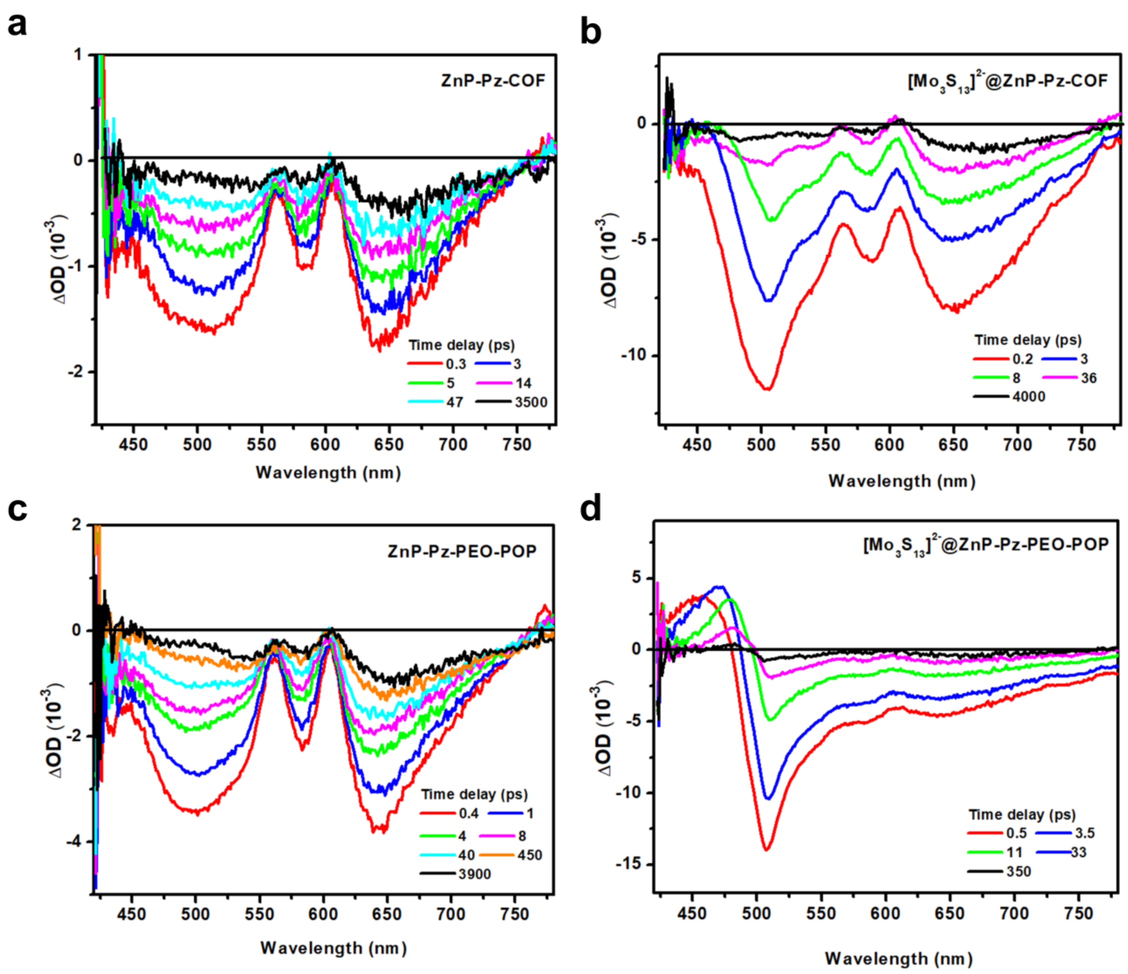

Supplementary Figure 22 | Femtosecond transient absorption spectra. a-d, Femtosecond transient absorption spectra of (a) ZnP-Pz -COF, (b) $[Mo_3S_{13}]^{2-}$@ZnP-Pz -COF and (c) ZnP-Pz –PEO-POP and (d) $[Mo_3S_{13}]^{2-}$@ZnP-Pz –PEO-POP, pumped at 400 nm.

3. Supplementary Tables

Supplementary Table 1 | Pawley refined crystal unit cell parameters of Zn-Pz-DHTP-COF and ZnP-Pz-PEO-COF

**COFs**	***a*** (Å)	***b*** (Å)	***c*** (Å)	***α***	***β***	***γ***
**ZnP-Pz-DHTP-COF**	25.6328	25.3789	4.8489	90°	90°	90°
**ZnP-Pz-PEO-COF**	25.6492	25.3858	4.6832	90°	90°	90°

Supplementary Table 2 | Atomistic coordinates for the AA-stacking mode of Zn-Pz-DHTP-COF using DFTB+ method

Atom	Element	*x*	*y*	*z*
C1	C	0.48881	1.68729	0.53083
C2	C	0.46114	1.71866	0.34174
C3	C	0.45765	1.77313	0.37785
C4	C	0.51423	1.76724	0.77528
C5	C	0.51497	1.71234	0.74969
C6	C	0.48487	1.79794	0.59085
H7	H	0.44154	1.70084	0.16731
H8	H	0.43497	1.79633	0.23376
H9	H	0.53505	1.78538	0.94467
H10	H	0.53651	1.68928	0.89931
C11	C	0.51527	0.19306	0.59012
C12	C	0.48588	0.22364	0.7751
C13	C	0.48512	0.27854	0.75058
C14	C	0.53937	0.27257	0.34394
C15	C	0.5427	0.21806	0.37825
C16	C	0.51148	0.30372	0.53296
H17	H	0.465	0.20541	0.94392
H18	H	0.46345	0.30151	0.90012
H19	H	0.55928	0.29062	0.17105
H20	H	0.56548	0.19499	0.23404
C21	C	0.49192	0.62836	0.49639
C22	C	0.44709	0.59836	0.47734
C23	C	0.54006	0.60433	0.47313
C24	C	0.58566	0.63333	0.46362
C25	C	0.62486	0.59856	0.45867
C26	C	0.60234	0.5491	0.44954
N27	N	0.54906	0.55136	0.45923
C28	C	0.39805	0.62099	0.47441
C29	C	0.36416	0.58202	0.42607
C30	C	0.39258	0.53575	0.42758
N31	N	0.44493	0.54474	0.45177
C32	C	0.5084	0.36262	0.49962
C33	C	0.46042	0.38655	0.46988
C34	C	0.55333	0.39258	0.48626
C35	C	0.60234	0.36989	0.48881
C36	C	0.63645	0.40887	0.44458
C37	C	0.60804	0.45513	0.44236
N38	N	0.55557	0.44617	0.46071
C39	C	0.41489	0.35753	0.4547
C40	C	0.37568	0.39223	0.44164

C41	C	0.39821	0.44174	0.43587
N42	N	0.45139	0.43954	0.45318
C43	C	0.63209	0.50384	0.43696
C44	C	0.36846	0.48701	0.42142
C45	C	0.30978	0.48359	0.42609
C46	C	0.69075	0.50751	0.4398
C47	C	0.71964	0.48297	0.6497
C48	C	0.77408	0.48483	0.65018
C49	C	0.80098	0.51374	0.44722
C50	C	0.77215	0.54009	0.24475
C51	C	0.71773	0.53599	0.2366
C52	C	0.28209	0.4553	0.22405
C53	C	0.22754	0.45231	0.2331
C54	C	0.1996	0.48027	0.43324
C55	C	0.22708	0.50832	0.63605
C56	C	0.28146	0.50874	0.63643
N57	N	0.14369	0.47744	0.43701
N58	N	0.48052	0.85403	0.61918
N59	N	0.51951	0.13694	0.6168
C60	C	0.5171	0.88466	0.71136
C61	C	0.11401	0.51558	0.52372
H62	H	0.59086	0.67558	0.45993
H63	H	0.66558	0.60943	0.46491
H64	H	0.38677	0.6616	0.50256
H65	H	0.32269	0.58782	0.40011
Zn66	Zn	0.50023	0.49546	0.45624
H67	H	0.61343	0.32922	0.51699
H68	H	0.678	0.40305	0.42272
H69	H	0.40981	0.31525	0.45245
H70	H	0.33499	0.38126	0.44071
H71	H	0.69992	0.46161	0.81264
H72	H	0.79409	0.4645	0.81436
H73	H	0.79204	0.56321	0.08929
H74	H	0.69653	0.55567	0.07305
H75	H	0.30281	0.43516	0.06035
H76	H	0.20696	0.42956	0.07866
H77	H	0.20661	0.52853	0.80007
H78	H	0.30162	0.52966	0.79942
N79	N	0.85697	1.51856	0.44198
C80	C	0.88983	1.4952	0.60548
H81	H	0.13063	0.55378	0.57094
C82	C	0.48325	1.10632	0.71295
C83	C	0.55055	0.9768	0.72789
C84	C	0.50836	0.94179	0.72494

N85	N	0.45897	0.96146	0.72689
C86	C	0.44981	1.01419	0.73103
C87	C	0.49199	1.0492	0.72532
N88	N	0.54139	1.02952	0.7242
C89	C	1.02555	1.55223	0.46889
C90	C	1.05693	1.50926	0.54423
C91	C	1.03299	1.46323	0.64803
C92	C	0.97862	1.46019	0.65923
C93	C	0.94691	1.50273	0.57929
C94	C	0.97114	1.54996	0.48793
H95	H	0.44529	1.12197	0.7673
H96	H	0.55534	0.86903	0.76042
H97	H	0.87657	1.46663	0.75493
H98	H	0.59031	0.96243	0.72606
H99	H	0.41005	1.02857	0.73173
H100	H	1.04363	1.58836	0.39804
H101	H	0.96095	1.42436	0.73668
O102	O	0.06124	0.42066	0.75562
O103	O	0.94372	0.59613	0.42383
H104	H	0.09887	0.42846	0.80157
H105	H	0.90602	0.59712	0.48099

Supplementary Table 3 | Atomistic coordinates for the AA-stacking mode of Zn-Pz-PEO-COF using DFTB+ method

**Atom**	**Element**	***x***	***y***	***z***
C1	C	0.48667	1.68912	0.57883
C2	C	0.45747	1.72024	0.38854
C3	C	0.45404	1.77471	0.42485
C4	C	0.51372	1.76948	0.82296
C5	C	0.51422	1.71456	0.79907
C6	C	0.48299	1.79988	0.63726
H7	H	0.43623	1.70102	0.20182
H8	H	0.42748	1.79895	0.28054
H9	H	0.53831	1.78971	0.99353
H10	H	0.53729	1.69037	0.96064
C11	C	0.5172	0.19519	0.63778
C12	C	0.48646	0.2256	0.82336
C13	C	0.48596	0.28053	0.79948
C14	C	0.5427	0.27482	0.38885
C15	C	0.54613	0.22035	0.42531
C16	C	0.51351	0.30596	0.57918
H17	H	0.4618	0.20553	0.99418
H18	H	0.46306	0.30521	0.95955
H19	H	0.5637	0.2946	0.20304
H20	H	0.5728	0.19639	0.28014
C21	C	0.49035	0.63019	0.54202
C22	C	0.44581	0.59967	0.52225
C23	C	0.53867	0.60679	0.51104
C24	C	0.58393	0.63624	0.49755
C25	C	0.62343	0.60196	0.48269
C26	C	0.60139	0.55231	0.46996
N27	N	0.54823	0.55397	0.48929
C28	C	0.39654	0.62169	0.51944
C29	C	0.36332	0.58268	0.45866
C30	C	0.3921	0.53668	0.45903
N31	N	0.44426	0.54617	0.48998
C32	C	0.50982	0.3649	0.54226
C33	C	0.46148	0.38832	0.51171
C34	C	0.55433	0.39541	0.52193
C35	C	0.60359	0.3734	0.51893
C36	C	0.63678	0.4124	0.45756
C37	C	0.60801	0.4584	0.45797
N38	N	0.55587	0.44892	0.48936
C39	C	0.41624	0.35886	0.49841
C40	C	0.37674	0.39313	0.48399

C41	C	0.39876	0.44279	0.47122
N42	N	0.45195	0.44114	0.49014
C43	C	0.63156	0.50741	0.44633
C44	C	0.36857	0.48767	0.44775
C45	C	0.30998	0.48322	0.43561
C46	C	0.69018	0.51183	0.43424
C47	C	0.72144	0.48622	0.63807
C48	C	0.7757	0.48844	0.62206
C49	C	0.80011	0.51905	0.41125
C50	C	0.76927	0.54756	0.21945
C51	C	0.71493	0.54257	0.22416
C52	C	0.28523	0.4525	0.22542
C53	C	0.2309	0.44753	0.22051
C54	C	0.20005	0.47602	0.41228
C55	C	0.22443	0.50664	0.62322
C56	C	0.27869	0.50883	0.63946
N57	N	0.1443	0.47185	0.39458
N58	N	0.47916	0.85604	0.66327
N59	N	0.52102	0.13904	0.66381
C60	C	0.51622	0.88665	0.75277
C61	C	0.11219	0.50874	0.4723
H62	H	0.58183	0.68109	0.50055
H63	H	0.66538	0.6168	0.48257
H64	H	0.39134	0.6655	0.5645
H65	H	0.3204	0.59178	0.41912
Co66	Co	0.50007	0.49755	0.48971
H67	H	0.60881	0.3296	0.56433
H68	H	0.67969	0.4033	0.41759
H69	H	0.41836	0.314	0.50119
H70	H	0.33479	0.3783	0.48417
H71	H	0.7025	0.46323	0.81962
H72	H	0.80046	0.46531	0.78067
H73	H	0.78836	0.57506	0.05793
H74	H	0.69069	0.56374	0.05544
H75	H	0.30948	0.43134	0.0567
H76	H	0.21152	0.42013	0.0595
H77	H	0.19987	0.52987	0.78227
H78	H	0.29762	0.53178	0.8212
N79	N	0.85584	1.52329	0.39421
C80	C	0.88792	1.48624	0.47025
H81	H	0.12786	0.54797	0.55382
C82	C	0.48382	1.10845	0.75203
C83	C	0.55023	0.97854	0.76963
C84	C	0.50785	0.94382	0.76444

N85	N	0.45862	0.9638	0.76309
C86	C	0.44977	1.01656	0.76639
C87	C	0.49215	1.05128	0.76401
N88	N	0.54139	1.0313	0.76584
C89	C	1.02366	1.54692	0.46189
C90	C	1.05493	1.50158	0.45868
C91	C	1.03139	1.45136	0.45353
C92	C	0.97643	1.44811	0.45525
C93	C	0.94516	1.49344	0.45754
C94	C	0.9687	1.54367	0.45957
H95	H	0.44483	1.12599	0.8199
H96	H	0.55514	0.86913	0.82206
H97	H	0.87221	1.44685	0.54945
H98	H	0.59171	0.96248	0.77698
H99	H	0.40811	1.03219	0.77106
H100	H	1.04253	1.58757	0.46648
H101	H	0.95649	1.40798	0.45478
O102	O	0.06366	0.40636	0.45848
C103	C	0.03886	0.35654	0.42738
C104	C	0.07927	0.31315	0.44055
O105	O	0.05456	0.26453	0.41046
C106	C	0.09379	0.22745	0.4567
C107	C	0.071	0.17256	0.43194
O108	O	0.11001	0.1353	0.47946
C109	C	0.08679	0.08726	0.41094
O110	O	0.93637	0.58861	0.47021
C111	C	0.9612	0.63866	0.45726
C112	C	0.92057	0.68181	0.47326
O113	O	0.94527	0.7307	0.46259
C114	C	0.90496	0.76731	0.48534
C115	C	0.92743	0.82247	0.47459
O116	O	0.88725	0.85922	0.49754
C117	C	0.91183	0.90789	0.46669
H118	H	0.0179	0.35493	0.21291
H119	H	0.00939	0.35085	0.60698
H120	H	0.10025	0.3147	0.65496
H121	H	0.1087	0.3187	0.26064
H122	H	0.11084	0.2331	0.67938
H123	H	0.1258	0.23278	0.29034
H124	H	0.0541	0.16687	0.20892
H125	H	0.03882	0.16738	0.59747
H126	H	0.10678	0.05427	0.53463
H127	H	0.09091	0.0797	0.17206
H128	H	0.04369	0.08853	0.47014

H129	H	0.98363	0.6422	0.24797
H130	H	0.98939	0.64279	0.64415
H131	H	0.89796	0.67817	0.6819
H132	H	0.89257	0.67794	0.2854
H133	H	0.88364	0.76137	0.6965
H134	H	0.8765	0.76168	0.30087
H135	H	0.94875	0.82839	0.2634
H136	H	0.95594	0.82803	0.65892
H137	H	0.91868	0.91615	0.23039
H138	H	0.95082	0.9072	0.58348
H139	H	0.88608	0.94009	0.56098

Supplementary Table 4 | Mo contents of samples based on ICP-AES results

Sample	$[Mo_3S_{13}]^{2-}$@ ZnP-Pz-PEO -COF	$[Mo_3S_{13}]^{2-}$@ ZnP-Pz-PEO-COF (low content sample)	$[Mo_3S_{13}]^{2-}$@ ZnP-Pz-PEO-COF (high content sample)	$[Mo_3S_{13}]^{2-}$@ ZnP-Pz-COF	$[Mo_3S_{13}]^{2-}$@ ZnP-Pz-DHT P-COF	$[Mo_3S_{13}]^{2-}$@ ZnP-Pz-PEO-POP	$[Mo_3S_{13}]^{2-}$/ ZnP-TP-PEO-COF
Mo (wt%)	5.61	3.22	8.27	6.21	5.55	5.48	4.94
Theoratical molar rato of Mo/Zn[1]	6	6	6	12	6	6	\
Determined molar rato of Mo/Zn	1.16	0.52	2.56	1.12	0.95	1.12	0.94

[1]Nitrogen atoms of pyrazine units are fully coordinated with $[Mo_3S_{13}]^{2-}$ clusters.

Supplementary Table 5 | Band bap structures

COFs	HOMO (eV)	LUMO (eV)	Eg_{elec} (eV)	Eg_{opt} (eV)
**ZnP-Pz-COF**	-5.25	-3.81	1.44	1.49
**ZnP-Pz-DHTP-COF**	-5.71	-3.83	1.88	1.51
**ZnP-Pz-PEO-COF**	-5.63	-3.87	1.76	1.45
**ZnP-TP-PEO-COF**	-5.71	-3.90	1.81	1.46
**ZnP-Pz-PEO-POP**	-5.34	-3.88	1.46	1.44
**$[Mo_3S_{13}]^{2-}$@ZnP-Pz-COF**	-5.25	-3.87	1.38	1.51
**$[Mo_3S_{13}]^{2-}$@ ZnP-Pz-DHTP-COF**	-5.73	-3.86	1.87	1.47
**$[Mo_3S_{13}]^{2-}$@ ZnP-Pz-PEO-COF**	-5.68	-3.87	1.81	1.46
**$[Mo_3S_{13}]^{2-}$/ ZnP-TP-PEO-COF**	-5.64	-3.95	1.69	1.48
**$[Mo_3S_{13}]^{2-}$@ ZnP-Pz-PEO-POP**	-5.24	-3.68	1.56	1.43

Supplementary Table 6 | Photocatalytic activity of typical COF-based photocatalysts

**Photocatalyst**	**Light**	**Reduction Catalyst**	**Sacrificial Donor**	**H_2 evolution Rate (mmol g^{-1} h^{-1})**	**Apparent Quantum Yield**	**Reference**
**ZnP-Pz-PEO-COF**	> 420 nm	$[Mo_3S_{13}]^{2-}$	Lactic acid	10.7	5.3% at 500 nm	This work
**[Co-1b]−COF**	Full spectra	Co complex	TEOA	0.163 (CH_3CN/H_2O=4/1)	–	8
**TpDTz COF**	Full spectra	Ni-thiolate cluster	TEOA	0.941	0.2% at 400 nm	9
**N_2-COF**	Full spectra	Co cluster	TEOA	0.782 (CH_3CN/H_2O=4/1)	0.16% at 400 nm	10
**Mo_3S_{13}@EB-COF**	> 420 nm	$[Mo_3S_{13}]^{2-}$ $Ru(bpy)_3Cl_2$ (photosensitizer)	Ascorbic acid	13.2 (DMF/H_2O=1/1); < 2 in pure H_2O system	4.49% at 475 nm	11
**ZnPor-DETH -COF**	> 400 nm	Pt	TEOA	0.413	0.32% at 450 nm	12
**PyTz-COF**	Full spectra	Pt	Ascorbic acid	2.072	–	13
**Tp-2C/BPy^{2+}-COF (19.10%)**	> 420 nm	Pt	Ascorbic acid	34.6	6.93% at 420 nm	14
**TtaTfa**	> 420 nm	Pt	Ascorbic acid	20.7	1.43% at 450 nm	15
**NKCOF-108**	> 420 nm	Pt	Ascorbic acid	11.6	2.96% at 520 nm	16
**TZ-COF-4**	> 420 nm	Pt	Ascorbic acid	4.296	1.3% at 420 nm	17

**BtCOF150**	≥ 400 nm	Pt	TEOA	0.75 ± 0.025	0.2% at 420 nm	18
***g*-$C_{54}N_6$-COF**	≥ 420 nm	Pt	TEOA	2.519	–	19
**FS-COF**	> 420 nm	Pt	Ascorbic acid	10.1	3.2% at 420 nm	20
**sp^2c-COF_{ERDN}**	≥ 420 nm	Pt	TEOA	2.12	0.48 % at 495 nm	21
***g*-$C_{40}N_3$-COF**	> 420 nm	Pt	TEOA	2.596	4.84% at 420 nm	22
***g*-$C_{18}N_3$-COF**	> 420 nm	Pt	Ascorbic acid	0.292	1.06% at 420 nm	23
**TP-BDDA**	≥ 395 nm	Pt	TEOA	0.324	1.8% at 520 nm	24
**N_3–COF**	≥ 420 nm	Pt	TEOA	1.703	–	25
**TFPT–COF**	Full spectra	Pt	TEOA	1.97	2.2% at 400 nm	26

Supplementary Table 7 | Fitting details of femtosecond transient absorption spectra at 500 nm

Sample	A_1	τ_1 (ps)	A_2	τ_2 (ps)	$\tau_{average}$* (ps)
ZnP-Pz-COF	0.06	2.7 ± 0.1	0.25	86 ± 7	70 ± 6
$[Mo_3S_{13}]^{2-}$ @ZnP-Pz-COF	1.13	4.28 ± 0.03	0.08	470 ± 30	35 ± 2
ZnP-Pz-PEO-COF	1.76	3.5 ± 0.2	0.23	180 ± 10	24 ± 1
$[Mo_3S_{13}]^{2-}$ @ZnP-Pz-PEO-COF	0.60	3.5 ± 0.2	0.40	2.1 ± 0.3	2.9 ± 0.2
ZnP-Pz-PEO-POP	0.54	2.4 ± 0.1	0.24	107 ± 7	35 ± 2
$[Mo_3S_{13}]^{2-}$ @ZnP-Pz-PEO-POP	0.57	4.18 ± 0.09	0.32	24 ± 1	11.3 ± 0.4

*The $\tau_{average}$ was calculated as weighted average.

4. Supplementary References

1. Yuasa, M., Oyaizu, K., Yamaguchi, A. & M. Kuwakado, Micellar cobaltporphyrin nanorods in alcohols. *J. Am. Chem. Soc.* **126**, 11128–11129 (2004).
2. Tsuda, A. et al. A molybdenum crown cluster forms discrete inorganic–organic nanocomposites with metalloporphyrins. *Angew. Chem., Int. Ed.* **43**, 6327–6331 (2004).
3. Li, J. et al. Pyrazine-fused isoindigo: a new building block for polymer solar cells with high open circuit voltage. *Chem. Commun.* **53**, 5882–5885 (2017).
4. Kuhnert, N., Rossignolo, G. M. & Periago, A. L. The synthesis of trianglimines: on the scope and limitations of the [3 + 3] cyclocondensation reaction between (1R,2R)-diaminocyclohexane and aromatic aicarboxaldehydes. *Org. Biomol. Chem.* **1**, 1157–1170 (2003).
5. Kretz, T., Bats, J. W., Lerner, H. W. & Wagner, T. Z. 2,5-Diformylbenzene-1,4-diol: a versatile building block for the synthesis of ditopic redox-active schiff base ligands. *Naturforsch. A.* **62b**, 66–74 (2007).
6. Kibsgaard, J., Jaramillo, T. F. & Besenbacher, F. Building an appropriate active-site motif into a hydrogen-evolution catalyst with thiomolybdate $[Mo_3S_{13}]^{2-}$ clusters. *Nat. Chem.* **6**, 248–253 (2014).
7. Lan, Z. et al. Reducing the exciton binding energy of donor–acceptor-based conjugated polymers to promote charge-induced reactions. *Angew. Chem., Int. Ed.* **58**, 10236–10240 (2019).
8. Gottschling, K. et al. Rational Design of Covalent Cobaloxime–Covalent Organic Framework Hybrids for Enhanced Photocatalytic Hydrogen Evolution. *J. Am. Chem. Soc.* **142**, 12146–12156 (2020).
9. Biswal, B. P. et al. Sustained Solar H_2 Evolution from a Thiazolo[5,4-d]thiazole-Bridged Covalent Organic Framework and Nickel-Thiolate Cluster in Water. *J. Am. Chem. Soc.* **141**, 11082–11092 (2019).
10. Banerjee, T. et al. Single-Site Photocatalytic H_2 Evolution from Covalent Organic Frameworks with Molecular Cobaloxime Co-Catalysts. *J. Am. Chem. Soc.* **139**, 16228–16234 (2017).
11. Cheng, Y. et al. Encapsulating $[Mo_3S_{13}]^{2-}$ Clusters in Cationic Covalent Organic Frameworks: Enhancing Stability and Recyclability by Converting a Homogeneous Photocatalyst to a Heterogeneous Photocatalyst. *Chem. Commun.* **54**, 13563-13566 (2018).

12. Chen, R. et al. Rational Design of Isostructural 2D Porphyrin-Based Covalent Organic Frameworks for Tunable Photocatalytic Hydrogen Evolution. *Nat. Commun.* **12**, 1354 (2021).
13. Li, W. Thiazolo[5,4-d]thiazole-Based Donor–Acceptor Covalent Organic Framework for Sunlight-Driven Hydrogen Evolution. *Angew. Chem., Int. Ed.* **60**, 1869-1874 (2021).
14. Mi, Z. et al. Covalent Organic Frameworks Enabling Site Isolation of Viologen-Derived Electron-Transfer Mediators for Stable Photocatalytic Hydrogen Evolution. *Angew. Chem., Int. Ed.* **60**, 9642-9649 (2021).
15. Yang, J. et al. Protonated Imine-Linked Covalent Organic Frameworks for Photocatalytic Hydrogen Evolution. *Angew. Chem., Int. Ed.* **60**, 19797-19803 (2021).
16. Zhao, Z. et al. Fabrication of Robust Covalent Organic Frameworks for Enhanced Visible-Light-Driven H_2 Evolution. *ACS Catal.* **11**, 2098–2107 (2021).
17. Wang, K. et al. Synthesis of Stable Thiazole-Linked Covalent Organic Frameworks via a Multicomponent Reaction. *J. Am. Chem. Soc.* **142**, 11131–11138 (2020).
18. Ghosh, S. et al. Identification of Prime Factors to Maximize the Photocatalytic Hydrogen Evolution of Covalent Organic Frameworks. *J. Am. Chem. Soc.* **142**, 9752–9762 (2020).
19. Xu, J. et al. Vinylene-Linked Covalent Organic Frameworks (COFs) with Symmetry-Tuned Polarity and Photocatalytic Activity. *Angew. Chem., Int. Ed.* **59**, 23845-23853 (2020).
20. Wang, X. et al. Sulfone-Containing Covalent Organic Frameworks for Photocatalytic Hydrogen Evolution from Water. *Nat. Chem.* **10**, 1180-1189 (2018).
21. Jin, E. et al. 2D sp^2 Carbon-Conjugated Covalent Organic Frameworks for Photocatalytic Hydrogen Production from Water. *Chem* **5**, 1632-1647 (2019).
22. Bi, S. et al. Two-Dimensional Semiconducting Covalent Organic Frameworks *via* Condensation at Arylmethyl Carbon Atoms. *Nat. Commun.* **10**, 2467 (2019).
23. Wei, S. et al. Semiconducting 2D Triazine-Cored Covalent Organic Frameworks with Unsubstituted Olefin Linkages. *J. Am. Chem. Soc.* **141**, 14272-14279 (2019).
24. Pachfule, P. et al. Diacetylene Functionalized Covalent Organic Framework (COF) for Photocatalytic Hydrogen Generation. *J. Am. Chem. Soc.* **140**, 1423-1427 (2018).
25. Vyas, V. S. et al. A Tunable Azine Covalent Organic Framework Platform for Visible Light-Induced Hydrogen Generation. *Nat. Commun.* **6**, 8508 (2015).
26. Stegbauer, L., Schwinghammer, K. & and Lotsch, B. V. A Hydrazone-Based Covalent Organic

Framework for Photocatalytic Hydrogen Production. *Chem. Sci.* **5**, 2789-2793 (2014).
