# Supplementary material for: Integrated interfacial design of covalent organic framework photocatalysts to promote hydrogen evolution from water
Source: Nat Commun. 2023 Jan 19;14:329. doi: 10.1038/s41467-023-35999-y (PMC9852592; doi:10.1038/s41467-023-35999-y)
Supplement: Supplementary file 2 — Description of Additional Supplementary Files [file 41467_2023_35999_MOESM2_ESM.pdf]

Description of Additional Supplementary Files

File Name: Supplementary Movie 1
Description: Supplementary Movie for ZnP-Pz-PEO-COF

File Name: Supplementary Movie 2
Description: Supplementary Movie for MO3S13@ZnP-Pz-PEO-COF
